# A 90-Day Oral Toxicity Study of an Ethanolic Root Extract of *Caesalpinia bonduc* (L.) Roxb. in Wistar Rats

**DOI:** 10.1155/2021/6620026

**Published:** 2021-01-27

**Authors:** Mariette Sindete, Adam Gbankoto, Razack Osseni, Nounagnon Darius Tossavi, Simon Azonbakin, Lamine Baba-Moussa, Anatole Laleye

**Affiliations:** ^1^Laboratory of Experimental Physiology and Pharmacology, Faculty of Sciences and Technology, University of Abomey-Calavi, Cotonou, Benin; ^2^Laboratory of Physiology, Faculty of Health Sciences, University of Abomey-Calavi, Cotonou, Benin; ^3^Laboratory of Histology, Reproductive Biology, Cytogenetics and Medical Genetics, Faculty of Health Sciences, University of Abomey-Calavi, Cotonou, Benin; ^4^Laboratory of Biology and Molecular Typing in Microbiology, Faculty of Sciences and Technology, University of Abomey-Calavi, Cotonou, Benin

## Abstract

**Background:**

Plant medicine is the oldest form of health care known to mankind; hence, studies on their safety for use are essential for the control of adverse drug effects. In Benin, *Caesalpinia bonduc* is one of many medicinal plants used as aphrodisiac, and for treatment of various ailments including prostatic hyperplasia. Despite its numerous ethnomedicinal benefits, toxicological information associated with its chronic use is currently limited.

**Objective:**

The present study therefore assessed the toxicity of an ethanolic root extract of *Caesalpinia bonduc* in Wistar rats.

**Methods:**

*Caesalpinia bonduc* root extract was administered by oral gavage at doses of 31.25, 125, and 500 mg/kg/day for 90 days to male Wistar rats, after which body weight changes, food consumption, urinary parameters, hematological and blood biochemical parameters, organ weights changes, gross pathology, and histopathology of vital organs were assessed.

**Results:**

There were no death or abnormal clinical signs, no significant changes in body weight gain or urinary parameters, and no changes in necropsy and histopathology findings of vital organs associated with extract treatment. However, some indices such as erythrocytes, total cholesterol, and aspartate amino transferase increased in rats treated with high doses of the extract, as well as relative weight of testes, followed by a decrease in food intake and prostate relative weight.

**Conclusion:**

The results indicate that an ethanolic root extract of *Caesalpinia bonduc* does not cause significant adverse effects and suggest its tolerability up to 500 mg/kg for daily administration of 90 days.

## 1. Introduction

The continued use of herbal medicines in traditional medicine is attributable not only to cultural and poverty reasons but also to its effectiveness against many diseases as they possess the ability to synthesize, through complex metabolic pathways, many bioactive compounds at different biological and pharmacological properties [1, 2]. So, in a quarter of biomedical medications regularly prescribed, at least one active ingredient comes from these plants and the rest of the substances are chemically produced in laboratories [3]. Therefore, the traditional herbal medicines are getting more and more significant attention in global population and the search for new natural drugs with fewer side effects, bringing an additional change in the modern world, is then highly appreciated [2, 4]. However, continued use of these plants for medication without validly reporting on their toxic profiles scientifically could negatively affect human health [2].

*Caesalpinia bonduc* (L.) Roxb. is one of the pantropical leguminous scandent shrubs which is studied by several authors for these multiple uses [5, 6]. This plant which belongs to the family of Caesalpiniaceae is commonly known as Fever Nut, Ayo, and Nicker Nut in English and Indian medicine system, Adjikoui in Benin Fon language, and Bonduc in French [7, 8]. All the parts of this plant have therapeutic value and have been used in the traditional system of medicine since ancient times [9]. *Caesalpinia bonduc* (CB) root is traditionally used in Benin as an aphrodisiac and in the treatment of prostatic adenoma [10, 11]. The tribal people of Andaman and Nicober Islands conventionally used the aqueous solution of the outer shell of the seeds of CB for the relief of symptoms of diabetes mellitus [12]. In the southwestern part of Nigeria, young twigs and leaves of CB are used as antimalarial medicinal decoction together with other medicinal plant [13]. In La Reunion and Madagascar, the roots are viewed as febrifuge and anthelmintic; they are mostly used as an astringent in leucorrhoea and blennorrhagia [14]. As scientifically validated pharmacological studies, recent studies show that ethanolic seed extracts of CB are effective in the prophylaxis management of asthma [15, 16]. The whole plant, the leaves, and the root bark are reported for their antidiabetic property [17, 18]. Isolated flavonoids from young twigs and leaves of CB have been reported to exhibit anticancer activity and high antioxidant capacity [19]. Researchers have reported the aphrodisiac property of aqueous and ethanolic extract of CB root as well as the acute and subacute (28 days) oral toxicity of ethanolic extract of CB root [20–22].

In low-income countries where most of the population engage in herbal medicine, the use of plants remains traditional [23]. But, the scientific knowledge about biological properties of active compounds of these plants as well as the doses to be administered is ignored and most of the time this situation leads to treatment failures [24]. It has been demonstrated that many traditional beneficial herbs have genotoxic or systemic (liver and kidney) toxicity [23]. Thus, it is necessary to conduct comprehensive safety analysis under strict guidelines for medicinal plants available. Our previous studies evaluated acute and subacute oral toxicity of ethanolic root extract of CB in Wistar rats and reported that it was safe to use [21]. In order to provide additional toxicological information about CB root, and help to guide optimization and validation of its traditional use, the present study was undertaken to assess the cumulative toxicity of CB root when orally administered once daily to Wistar rats for a period of 90 days.

## 2. Materials and Methods

### 2.1. Plant Material

*Caesalpinia bonduc* roots were harvested in May, 2018, from a field in the area of Sèhouè (6°55′54″ N - 2°16′27″ E), located in the Atlantique Department of Southern Benin. The samples were authenticated at the National Herbarium of the University of Abomey-Calavi, Benin, where voucher specimen was cataloged under number AA6743/HNB. The roots were washed with tap water and distilled water to remove the sand and any dirt before being cut into very thin slices and then dried at 25°C until the complete drying. The dried roots were ground into powder using electric grinder (Flour Mills Nigeria, El Motor N°1827).

### 2.2. Preparation of Plant Extract

Fifty (50) grams of powder was macerated in 500 ml of ethanol 95% into the enclosed glass jars for seventy-two hours under orbital shaker (VWR® Advanced Orbital Shaker, Model 5000, USA), 180 rpm at 25°C. The macerate obtained was filtered on the cotton and then on Whatman N°1 (filter paper, London). The filtrate was collected into glass conical flask and concentrated in a rotary evaporator (VWR® IKA, RV8, USA) at 40°C under reduced pressure. The concentrate was oven-dried at 40°C to obtain the dry ethanol extract which was stored at 4°C until being used. The crude extract was reconstituted in dimethyl sulfoxide (DMSO) to afford various concentrations used in this study whenever needed.

### 2.3. Experimental Animals

Twenty-four (24) male Wistar rats (*Rattus norvegicus*), 13 weeks old, weighing between 200 and 220 g were used to perform the study. The animals were maintained in the animal facility located within the Faculty of Health Sciences, University of Abomey-Calavi, Benin, under the housing conditions of temperature: 25 ± 2°C and photoperiod: 12 h light and dark in standard cages with top grill. All animals were individually marked by a temporary marking of the tail in indelible ink and assigned based on their body weights into four experimental groups of six rats each as indicated below in experimental design. Paddies husks were used as the bedding materials that were cleaned on daily basis. Except for overnight food and water deprivation before blood and urine sampling, the animals were allowed free access to rat pellets (commercial complete food) which consisted of 18% crude protein, 1.16% calcium, 0.80% phosphorus, 0.82% lysin, 14.4% crude fiber, 7.12% crude fat, and tap water ad libitum during the experiment period. The experiments were approved by the Ethic Committee of Research of Applied Biomedical Sciences Institute, Abomey-Calavi University, Benin. Care and handling of experimental animals were performed under well-founded conditions in compliance with the guidelines of European convention for protection of vertebrate animals and other scientific purposes [25] and in line with the applicable guidelines for repeated dose 90-day oral toxicity studies of OECD [25, 26].

### 2.4. Experimental Design

A 90-day oral repeated-dose toxicity was conducted according to Organization for Economic Cooperation and Development (OECD) test guideline 408 for testing chemicals [25] repeated by researchers [27–31]. CB root extract was administered once daily by oral gavage at 31.25, 125, or 500 mg/kg for 90 consecutive days. Dose levels were selected based on results from the four-week repeat dose studies [21, 32]. Control group was treated with DMSO, the vehicle used to dissolve CB root extract.

### 2.5. General Observations, Food Consumption, and Body Weight

Mortality, signs of illness, behavioural changes, and development of any clinical signs during the course of the experiment were observed daily and recorded. The food intake was also recorded daily, by subtracting the quantity of food remaining from that served. Body weight (BW) was recorded the day before CB root extract administration and weekly during the experimental period. After 90 days of treatment and prior to scheduled necropsy, the final body weight of animals was recorded and the body weight gain was calculated as (Final BW − Initial BW/Initial BW) × 100 [33].

### 2.6. Hematology, Serum Biochemistry, and Urinalysis

At the end of the 90-day experimental period, all animals were housed individually in metabolism cages and fasted overnight (16 h) for urine collection. The urine sample was collected in graduated tubes (50 ml urine container, China), positioned at the bottom of the cages. Specific gravity, pH value, glucose, ketone, protein, bilirubin, urobilinogen, nitrite, occult blood, and leukocyte were determined with the test strips (DUS™ 10, Standard Diagnostic, Korea) whereas urine color and appearance were evaluated visually. After urine collection, all animals were anaesthetized by intraperitoneal injection of sodium thiopental (50 mg/kg); the anaesthesia depth was checked by a pinch stimulus applied gently to the hind paws of the animal. Blood samples were then collected by retro-orbital puncture using hematocrit micro tubes for hematological (ethylene diamine tetra-acetic acid tubes) and biochemical (non-heparinized tubes) analysis. Hematological parameters included erythrocytes, hemoglobin (HGB), hematocrit, mean globular volume (MGV), mean corpuscular hemoglobin (MCH), mean corpuscular hemoglobin concentration (MCHC), leucocytes, neutrophil, lymphocyte, monocyte, eosinophil, and platelets. For serum biochemical analyses, blood samples without additive were left at 25°C for 1 h and then centrifuged after coagulation at 3000 rpm for 15 minutes. The sera were collected using a micropipette and transferred into labelled Eppendorf tubes (Eppendorf, microcentrifuge tube, Nigeria), and then stored in a −80°C freezer until analyses. Biochemistry parameters included alanine aminotransferase (ALT), aspartate aminotransferase (AST), total protein, glucose, blood urea nitrogen, creatinine, total cholesterol, sodium, potassium, and chloride.

### 2.7. Gross Observation and Organ Weights

After exsanguination, each rat was sacrificed by cervical dislocation under sodium thiopental (50 mg/kg) anaesthesia and then dissected for the macroscopic examination of the cranial and thoracic cavities, abdominal cavity, and all orifices as reported by researchers [34–36]. The liver, kidney, testis, prostate, heart, and stomach were isolated and freed from the adhering connective tissues and rinsed in 0.9% sodium chloride solution prior to being weighted for absolute weight determination. Relative organ weight was calculated based on the following equation: relative weight = (weight of the organ/body weight of the animal on sacrifice day) × 100 [37, 38].

### 2.8. Histopathological Study

Each isolated organ was fixed in 10% formalin solution [39]. The fixed testes were dehydrated in increasing concentration of alcohol baths (50, 70, 80, 90, and 100%) and then cleared in xylene. After incubation of paraffin in a 60°C incubator, they were embedded and blocked in paraffin wax at the same temperature and then freezed. The paraffin blocks were cut into sections of 5 *μ*m thickness using a microtome and then mounted on gelatinous water coated slides and dried. For hematoxylin and eosin (HE) staining, the sections on the slides were dewaxed with xylene and hydrated in a decreasing concentration of alcohol followed by water bathing before being stained with hematoxylin, ammonia complex blue, and eosin solutions. The sections were washed in running tap water after each staining step. After clearing up in the xylene, the slides were permanently mounted under a coverslip using Eukitt mounting resin and examined under light microscope (Olympus, Japan) for structural changes and photomicrographs were taken.

### 2.9. Statistical Analysis

All values were presented as means ± SEM (standard error of mean). The data were analyzed using one-way analysis of variance (ANOVA) followed by Tukey's multiple comparisons test. *p* value ≤0.05 was considered to be statistically significant. Statistical analyses were performed using GraphPad Prism software version 6 (GraphPad Software Inc., San Diego, USA).

## 3. Results

### 3.1. Clinical Observations

There were no clinical changes in extract-treated rats compared with control group; all rats survived and appeared healthy. Physical examinations did not show any treatment-related adverse effects and no abnormal behaviors were discovered in rats treated with CB root extract at different concentrations during the 90-day period.

### 3.2. Effects on Body Weight and Food Consumption

**Figure 1:**
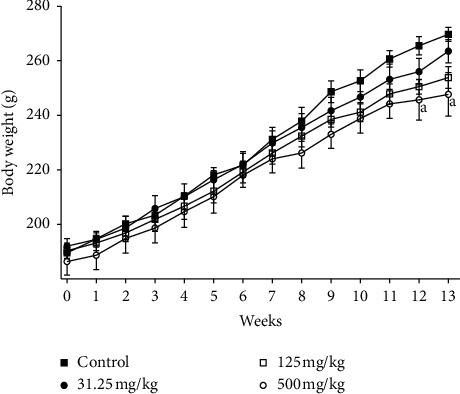
Effects of ethanolic root extract of *Caesalpinia bonduc* on body weight of rats after 90-day oral treatment. Values are means ± SEM. “a” indicates significance (*p* ≤ 0.05) from control group.

**Figure 2:**
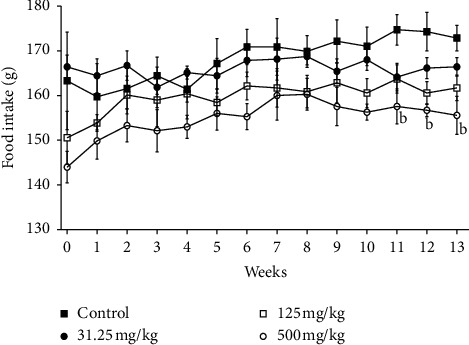
Effects of ethanolic root extract of *Caesalpinia bonduc* on food intake of rats after 90-day oral treatment. Values are means ± SEM. “b” indicates significance (*p* ≤ 0.01) from control group.

**Figure 3:**
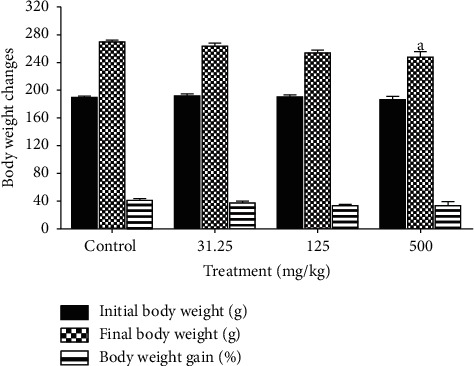
Effects of ethanol extract of *Caesalpinia bonduc* root on body weight changes of rats after 90-day oral treatment. Values are means ± SEM. “a” indicates significance (*p* ≤ 0.05) from control group.

Significant decrease (*p* ≤ 0.05) in body weight was noticed by weeks 12 and 13 of treatment in rats receiving 500 mg/kg of CB root extract when compared to the control group (Figure 1). That is in line with significant decrease (*p* ≤ 0.01) of food intake recorded at the same dose in these rats (Figure 2). Despite the decrease of final body weight, no differences in body weight gain were recorded compared to the control group (Figure 3); all treated animals gained weight. No changes (*p* ≥ 0.05) were found in body weight, body weight gain, and food consumed between the groups receiving 31.25 and 125 mg/kg CB root extract and control group.

### 3.3. Effects on Urinary Parameters

After 90-day administration of ethanolic root extract of CB, no notable changes (*p* > 0.05) were recorded for the urinary parameters of the treated groups in comparison with the control group. The nitrite, bilirubin, ketones, and glucose were absent in the urine and the urobilinogen level was normal for all rats; all parameters were within normal limits. A clear appearance of urine was observed in all groups and no significant difference was observed between the control and treated groups for the urine volume (Table 1).

### 3.4. Effects on Hematological and Serum Biochemical Parameters

All hematological parameters showed normal values in treated groups except neutrophils level that decreased significantly (*p* ≤ 0.05) in rats treated with 31.25 mg/kg of CB root extract compared to control group as well as increase of erythrocytes levels in rats treated with 500 mg/kg. However, both the mean neutrophil and the mean of erythrocytes were within the normal range. In 125 mg/kg treated group, no adverse effect of hematological parameters was recorded as compared with the control (Table 2). The serum biochemical parameters are presented in Table 3. The examination of renal function during the toxicity test was conducted by examining the urea, creatinine, and electrolytes levels; in comparison with the control group, no significant differences were discovered in the CB root treated groups for these parameters. However, a dose-dependent increase in aspartate amino transferase (AST) activity, which is significant (*p* ≤ 0.05) in 125 and 500 mg/kg treated group, was recorded. In comparison with the control group, CB root extract (500 mg/kg) increased significantly the levels of total cholesterol (*p* ≤ 0.05) and decreased those of glucose.

### 3.5. Effects on Absolute and Relative Organ Weights

**Figure 4:**
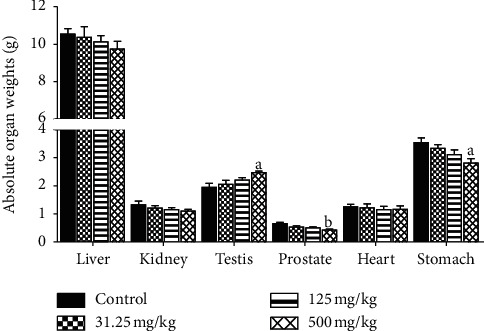
Effects of ethanol extract of *Caesalpinia bonduc* root on absolute organ weights of rats after 90-day oral treatment. Values are means ± SEM. “a” or “b” indicates significance (*p* ≤ 0.05 or *p* ≤ 0.01, respectively) from control group.

**Figure 5:**
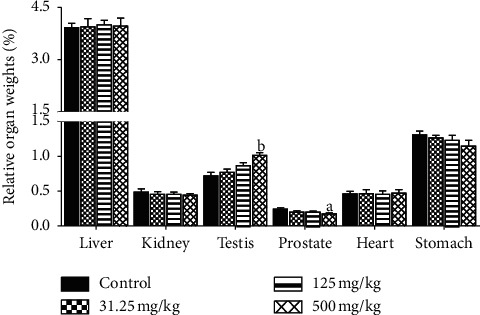
Effects of ethanol extract of *Caesalpinia bonduc* root on relative organ weights of rats after 90-day oral treatment. Values are means ± SEM. “a” or “b” indicates significance (*p* ≤ 0.05 or *p* ≤ 0.01, respectively) from control group.

The absolute weight of testis was significantly increased (*p* ≤ 0.05) in rats treated with 500 mg/kg of CB root extract as well as decreased prostate absolute weight (*p* ≤ 0.01) and stomach (*p* ≤ 0.05) weight in comparison with control group. Between the treated groups with 31.25 and 125 mg/kg and control, there were no significant differences in organ weights (Figure 4). In the same way, significant decrease (*p* ≤ 0.05) in the relative weight of prostate and significant increase (*p* ≤ 0.01) in the relative weight of testis were observed in 500 mg/kg treated group. Other treated groups did not show any differences versus the control (Figure 5).

### 3.6. Effect on Gross Observations

Gross examinations in organs indicated that no pathological abnormalities were detected between treatment and control groups. No inflammation, internal bleeding, lesions, or deformity was observed in the liver, kidney, testis, prostate, heart, and stomach in treated rats compared to control group. No presence of nodules and no abnormal macroscopic changes in color, size, shape, texture, atrophy, or hypertrophy were observed compared with the control following administration of CB root extract at different doses for 90 days of treatment.

### 3.7. Effect on Organs Histology

**Figure 6:**
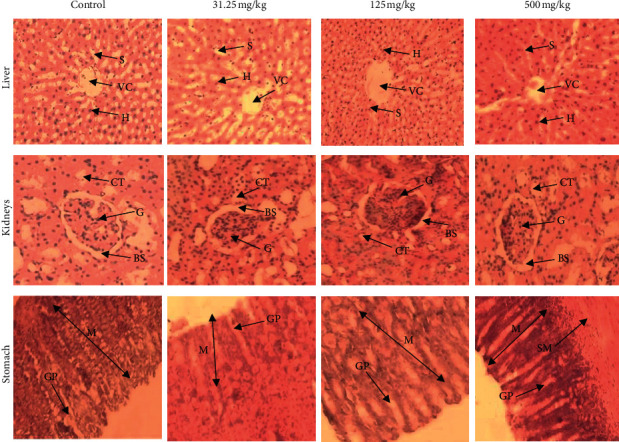
Representative photomicrographs of liver, kidney, and stomach of rats after 90-day oral administration of ethanolic root extract of *Caesalpinia bonduc*. Hematoxylin and eosin staining; magnification: ×200. Central vein (CV), hepatocytes (H), sinusoids (S), glomerulus (G), Bowman's space (BS), convoluted tubules (CT), mucosa (M), submucosa (SM), gastric pits (GP).

Histopathological analysis revealed no alterations of the liver, kidney, testis, prostate, heart, and stomach from animals; no adverse histopathological changes were observed. The liver sections of control group and the three experimental groups showed normal histological architecture of central vein (CV) and hepatocytes (H) with visible nuclei arranged as radial plates around the central vein and sinusoids (S); no tubular congestion, no cytoplasmic inclusions, and no inflammation were recorded (Figure 6). The kidney sections of control and experimental groups showed normal histological structure of glomerulus (G), Bowman's space (BS), and convoluted tubules (CT), and no glomerular congestion or inflammation (Figure 6).

**Figure 7:**
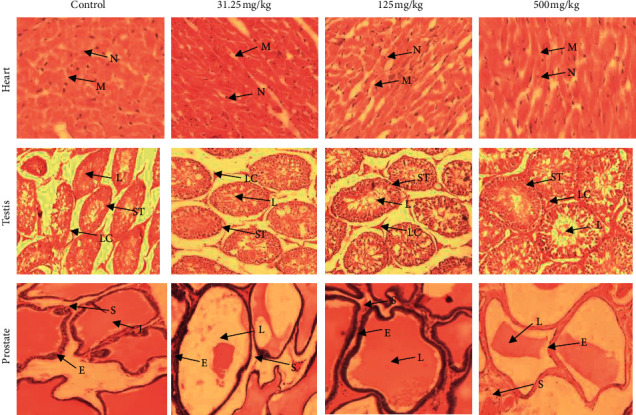
Representative photomicrographs of heart, testis, and prostate of rats after 90-day oral administration of ethanolic root extract of *Caesalpinia bonduc*. Hematoxylin and eosin staining; magnification: ×200. Nuclei (N) of cardiomyocytes, myofibers (M), seminiferous tubules (ST), lumen (L), Leydig cells (LC), epithelial cells (E), stroma (S).

Cross section of rats' stomach of all groups showed normal mucosa (M) with visible gastric pits (GP) in good condition and submucosa (SM). No structural abnormality was detected. Compared with the control group, CB root extract administration did not induce gastric damage, with no disruption of the superficial region of gastric gland, and no mucosa cell loss (Figure 6). The heart section of control and experimental groups showed normal histological structure of nuclei (N) of cardiomyocytes and myofibers (M). No obvious damage and no focal myocarditis were observed. There was a presence of muscle fibers organized into bundles, disks which unite the adjacent cardiac cells with the presence of several layers of muscle cells (Figure 7).

The testis sections of control and experimental groups showed normal histological architecture of seminiferous tubules (ST) and lumen (L) filled with spermatozoa and Leydig cells (LC) grouped in colony between the seminiferous tubules. But, well-developed seminiferous tubules were observed in treated rats at 500 mg/kg group. No obvious damage was observed. Interstitial space was also maintained with absence of apoptotic cells (Figure 7). The prostate sections of control and experimental groups of rats showed normal histological architecture of prostatic tissue with acini of different sizes which consisted of a layer of epithelial cells (E), stroma (S) between acini, and large lumen (L); no obvious disruption of the histoarchitecture, no irregular acinar folding, and no intraluminal projections were observed in CB root extract treated rats (Figure 7).

## 4. Discussion

Parameters such as general behavior, mortality, food consumption, and body weight change are the main indices considered in the evaluation of first signs of toxicity in rodents [40, 41]. Determination of food consumption is important to study the safety of a product with therapeutic purpose as proper intake of nutrients is essential to the physiological status of the animal and give a good impression of the appropriate response to the treatment. Body weight changes are an indicator of adverse side effects, and losing more than 20% of the animal body weight is regarded as critical and has been defined as one of the humane endpoints in several international guidelines [42]. There were no observable signs of toxicity or mortality in CB root extract-treated rats, which demonstrates its safety. There were no significant changes in body weight gain relative to control, except final body weight that was significantly affected in 500 mg/kg treated rats at the end of treatment period. This decreasing is in compliance with the food intake lowering noticed. This fact may be related to the proprieties of certain secondary metabolites to inhibit appetite and consequently reducing body weight such as triterpenes and saponins [43].

Our previous studies showed the presence of flavonoids, alkaloids, tannins, saponins, free anthracenics, quinoid derivatives, phenolics, coumarins, anthocyans, leucoanthocyans, triterpenoids, steroids, and mucilages in CB root [20, 22]. Studies confirmed that herbal extracts, a mixture of Scutellariae radix and Platycodi radix, containing the active ingredients baicalin and saponin were able to decrease body weight in rats [44]. Other researchers reported that platycodin D, a major saponin component of Platycodi radix, inhibits fat accumulation and adipogenesis [45]. According to Dai et al. [28], it is generally accepted that a decrease of 10% body weight gain of treated rats, when compared to the control rats, is defined as toxicological significance. However, the differences in body weight and food consumption observed with respect to control in this study were minor, less than 10%, and therefore considered to be without toxicological relevance.

Organ weight could be a meaningful indicator of treatment-related changes with or without corresponding histopathological examination in repeated toxicity studies [46]. It can provide direct evidence for pathological changes and can also indicate organs influenced by drugs; it is also an indicator of atrophy and hypertrophy [41]. However, relative weights of liver, kidneys, heart, and stomach in all treated groups did not reveal significant difference relative to the control group except the testis and prostate of animals treated with 500 mg/kg of CB root extract. This observation is consistent with the absolute testis and prostate weights in this group and considered to be without toxicological relevance because there were no related histological findings. On the other hand, the assessment of histopathological alterations in liver, kidneys, heart, stomach, testis, and prostate, which are vital organs, was considered as a basic test in the safety assessment of products. An improvement in the histoarchitecture of the testis and the prostate was noted in the 500 mg/kg treated rats compared to the control rats and the treated rats with the 31.25 and 125 mg/kg of CB root extract. It should be noted that the treatment duration could also influence the absolute organ weight [47], because researchers revealed no significant difference in the absolute organ weight of rats after acute toxicity study of ethanol extract of CB root [21]. These results are in agreement with those of [47] that reported no significant changes in relative organ weight of animals treated with methanol extract of *Caesalpinia bonducella* at 100 and 200 mg/kg. This is also in agreement with published works by [48].

Hematological and clinical chemistry parameters are good indicators in determining of toxic effects of plants and substances [49]; they are appropriated to risk evaluation as the hematological system has a higher prognostic value for toxicity [42]; it is an excellent index of adverse effects on hematopoietic system which is one of the most sensitive targets for toxic compounds [50]. Changes in the hematopoietic system have a higher predictive value for human toxicity when data are translated from animal studies [51]; it is regarded as a critical index of the physiological and pathological status of humans and animals [52]; it reflects the status of bone marrow activity and intravascular effects such as hemolysis and anemia [52]. In this study, administration of ethanol extract of CB root in rats for a period of 90 days produced no significant change in blood parameters except an increase in erythrocytes and decrease in neutrophil. This fact may be attributed to the presence of alkaloids in CB root which are responsible for stimulating the erythropoiesis system according to [53].

Evaluation of kidney and liver function is important in the assessment toxicity of plant extracts as both of them are necessary for the survival of organism [42]. Blood creatinine, urea, uric acid, and renal clearance remain semiotic parameters for diagnosis of renal function [41]. An increase in the level of these parameters in the blood is associated with reduced renal function and increased renal failure [41]. Creatinine, the most widely used biomarker for renal injury assessment, is a residual product of creatine produced endogenously at a practically constant rate and released into body fluids; it is freely filtered by the glomeruli and is not reabsorbed in renal tubules [54]. Urea, the main metabolite derived from protein degradation, is 90% excreted by the kidneys and also used as a marker of renal function [55]. Plasma elevations of these markers provide evidence of renal overload and acute renal failure [56]. Urea clearance falls as the kidney fails and as a result, urea tends to accumulate in diseased kidneys that are unable to excrete these substances at a normal rate; this will raise blood urea level [39]. In this study, no differences were observed in creatinine and urea levels when compared to the control group. These findings were supported by histopathological examinations of the kidneys, showing normal architecture of the histological structure.

The abnormal elevation of the liver enzymes (ALT and AST) is usually associated with liver damage or alteration in bile flow [57]. Alanine aminotransferase (ALT) is a cytosolic enzyme secreted primarily by liver cells and released into the blood stream when these cells are damaged [44]. It is a liver-specific enzyme, the most sensitive marker for liver cell damage, indicator of hepatotoxicity [44]. Aspartate aminotransferase (AST) is found primarily in the red blood cells, cardiac and skeletal muscles, lungs, and kidney; it is not specific to liver as ALT [57]. A significant increase in aspartate aminotransferase (AST) activities in serum for the 125 mg/kg treated group and 500 mg/kg treated group was recorded. Therefore, it is meaningful to investigate the effects of CB root extract on liver histology. Liver histology was assessed by using the hematoxylin and eosin (HE) staining. Histological analysis revealed no hepatocyte abnormalities. The treatment did not cause liver injury in rats as indicated by a significant increase in AST. These changes in AST caused by CB root are minor and considered unlikely to represent hepatic injury. ALT is the most sensitive marker of liver damage and toxicity since AST is also found in abundance in kidneys, testes, and cardiac and skeletal muscles which may explain its increase in this study [43, 51].

The liver and kidneys have fundamental roles in the metabolism and excretion of drugs or plant products [39]. Exogenous chemicals and their metabolites might result in toxicity or cell damage on these organs [57]. Therefore, the assessment of histopathological alterations in these organs is considered as a basic test in the safety assessment of tested materials [42]. In this study, rats treated with doses of 31.25, 125, and 500 mg/kg of ethanol extract of CB root showed no change in the histopathological structure of the liver, kidney, testis, prostate, heart, and stomach. The general architecture of the vital organs is normal as compared with controls; there were no toxic changes confirming the safety of the extract on the vital organs. The results of our research confirm that there are no adverse effects due to the administration of CB root in rats. Toxicity studies in animals often deliver valued evidence to predict adverse effects of new drugs in humans [49].

## 5. Conclusion

Ethanolic root extract of *Caesalpinia bonduc* did not produce adverse effects in male Wistar rats at treatment doses. It is well tolerated up to the dose of 500 mg/kg administered daily for 90 days. The extract did not cause any morbidity or lethality or produce any important serum biochemistry alteration, hematological or histopathological damage, which demonstrates its safety of use.

## Figures and Tables

**Table 1:** Effects of ethanolic root extract of *Caesalpinia bonduc* on urinary parameters of rats after 90-day oral treatment.

Parameters	Observations	Control	Repeated dose (mg/kg)		
			31.25	125	500
Number of rats		6	6	6	6

Ketone body (mg/dL)	Negative	6	6	6	6
	±5	0	0	0	0
	+15	0	0	0	0

Bilirubin	Negative	6	6	6	6
	+	0	0	0	0
	++	0	0	0	0

Occult blood (RBC/*μ*L)	Negative	6	6	6	6
	Trace	0	0	0	0
	+25	0	0	0	0

Protein (mg/dL)	Negative	0	0	0	0
	+30	4	3	2	0
	++100	2	3	3	4
	+++300	0	0	1	2

pH	Less than 6	2	0	0	0
	6–8	4	6	6	6
	More than 8	0	0	0	0

Leukocytes (WBC/*μ*L)	Negative	6	6	6	6
	Trace	0	0	0	0
	+70	0	0	0	0

Urobilinogen (mg/dL)	0.1	6	6	6	6
	1	0	0	0	0
	2	0	0	0	0

Glucose (mg/dL)	Negative	6	6	6	6
	±100	0	0	0	0

Specific gravity	≤1.010	2	3	0	0
	1.015	4	0	4	2
	1.020	0	2	2	1
	1.025	0	1	0	3
	≥1.030	0	0	0	0

Nitrite (±)	Negative	6	6	6	6
	Trace	0	0	0	0
	Positive	0	0	0	0

Appearance	Clear	6	6	6	6

Color	Yellow	0	6	0	0
	Dark yellow	6	0	0	0
	Pale yellow	0	0	0	6
	Amber	0	0	6	0

Volume (ml/16 h)		5.77 ± 0.54	6.16 ± 0.30	5.45 ± 0.43	6.20 ± 0.22

**Table 2:** Effects of ethanolic root extract of *Caesalpinia bonduc* on hematological parameters of rats after 90-day oral treatment.

Parameters	Control	31.25 mg/kg	125 mg/kg	500 mg/kg
Hemoglobin (g/dL)	16.27 ± 0.27	14.57 ± 0.50	15.70 ± 0.56	18.02 ± 0.43
Hematocrit (%)	58.50 ± 1.06	61.33 ± 2.65	55.67 ± 2.55	53.50 ± 3.00
Erythrocytes (T/L)	7.65 ± 0.40	6.40 ± 0.22	8.26 ± 0.20	9.19 ± 0.43^a^
MGV (fL)	72.67 ± 2.24	68.83 ± 2.50	66.50 ± 1.73	70.33 ± 1.80
MCH (pg)	22.83 ± 1.25	25.17 ± 1.14	23.50 ± 1.00	20.00 ± 0.73
MCHC (%)	30.50 ± 1.31	27.17 ± 2.30	32.67 ± 1.61	29.33 ± 1.10
Leucocytes (G/L)	11.88 ± 0.50	10.43 ± 0.40	11.25 ± 0.60	12.58 ± 1.00
Neutrophil (G/L)	5.82 ± 0.56	4.14 ± 0.26^a^	6.42 ± 0.33	5.40 ± 0.31
Eosinophil (G/L)	0.58 ± 0.05	0.42 ± 0.08	0.63 ± 0.06	0.56 ± 0.07
Monocyte (G/L)	0.67 ± 0.08	0.92 ± 0.06	0.75 ± 0.04	0.82 ± 0.14
Lymphocyte (G/L)	4.83 ± 0.17	5.64 ± 0.41	5.30 ± 0.30	6.54 ± 1.00
Platelet (10^3^/*μ*L)	647.30 ± 27.57	567.20 ± 23.40	692.30 ± 23.50	725.50 ± 18.00

Values are mean ± SEM, mean globular volume (MGV), mean corpuscular hemoglobin (MCH), mean corpuscular hemoglobin concentration (MCHC). “a” indicates significance (*p* ≤ 0.05) from control group.

**Table 3:** Effects of ethanolic root extract of *Caesalpinia bonduc* on biochemical parameters of rats after 90-day oral treatment.

Parameters	Control	31.25 mg/kg	125 mg/kg	500 mg/kg
Glucose (G/L)	0.62 ± 0.06	0.51 ± 0.03	0.49 ± 0.02	0.46 ± 0.05^a^
Urea (G/L)	0.71 ± 0.06	0.62 ± 0.05	0.58 ± 0.03	0.66 ± 0.02
Creatinine (mg/L)	6.22 ± 0.35	6.05 ± 0.50	5.63 ± 0.20	5.45 ± 0.40
Total cholesterol (G/L)	0.67 ± 0.04	0.71 ± 0.03	0.73 ± 0.05	0.81 ± 0.03^a^
Total protein (G/L)	37.15 ± 0.55	38.07 ± 1.00	40.51 ± 1.25	40.97 ± 0.52
AST (UI/L)	150.50 ± 2.30	158.70 ± 3.00	163.80 ± 3.40^a^	171.30 ± 4.00^a^
ALT (UI/L)	86.33 ± 4.04	75.67 ± 2.01	78.67 ± 3.50	95.17 ± 4.00
Sodium (mEq/L)	141.20 ± 3.70	143.00 ± 3.00	137.20 ± 4.40	139.50 ± 3.37
Potassium (mEq/L)	3.80 ± 0.14	4.13 ± 0.32	4.56 ± 0.40	4.76 ± 0.50
Chloride (mEq/L)	108.70 ± 1.20	107.50 ± 1.60	109.20 ± 0.70	108.30 ± 1.05

Values are mean ± SEM, aspartate aminotransferase (AST), alanine aminotransferase (ALT). “a” indicates significance (*p* ≤ 0.05) from control group.

## Data Availability

The data generated and used for this study are available from the corresponding author upon request.
